# An Updated Review of Pemphigus Diseases

**DOI:** 10.3390/medicina57101080

**Published:** 2021-10-09

**Authors:** Ali M. Malik, Sarah Tupchong, Simo Huang, Abhirup Are, Sylvia Hsu, Kiran Motaparthi

**Affiliations:** 1 College of Medicine, University of Florida, Gainesville, FL 32606, USA; ali.malik@ufl.edu (A.M.M.); acare2019@hotmail.com (A.A.); 2 Lewis Katz School of Medicine, Temple University, Philadelphia, PA 19140, USA; sarah.tupchong@temple.edu; 3 Department of Dermatology, Lewis Katz School of Medicine, Temple University, Philadelphia, PA 19140, USA; simo.huang@tuhs.temple.edu (S.H.); sylvia.hsu@tuhs.temple.edu (S.H.); 4 Department of Dermatology, College of Medicine, University of Florida, Gainesville, FL 32606, USA

**Keywords:** pemphigus dermatoses, pemphigus vulgaris, pemphigus foliaceus, IgA pemphigus, pemphigus

## Abstract

Clinicians may encounter a variety of skin conditions that present with vesiculobullous lesions in their everyday practice. Pemphigus vulgaris, pemphigus foliaceus, IgA pemphigus, and paraneoplastic pemphigus represent the spectrum of autoimmune bullous dermatoses of the pemphigus family. The pemphigus family of diseases is characterized by significant morbidity and mortality. Considering the risks associated with a delayed diagnosis or misdiagnosis and the potential for overlap in clinical features and treatment, evaluation for suspected pemphigus disease often requires thorough clinical assessment and laboratory testing. Diagnosis is focused on individual biopsies for histopathology and direct immunofluorescence. Additional laboratory methods used for diagnosis include indirect immunofluorescence and enzyme-linked immunosorbent assay. Recent advancements, including anti-CD20 therapy, have improved the efficacy and reduced the morbidity of pemphigus treatment. This contribution presents updates on the pathophysiology, clinical features, diagnostic work-up, and medical management of pemphigus. Improved strategies for diagnosis and clinical assessment are reviewed, and newer treatment options are discussed.

## 1. Introduction

Pemphigus refers to a family of rare acantholytic autoimmune dermatoses of the mucocutaneous membranes in which acantholysis, or the loss of cell-to-cell adhesion, causes potentially lethal bullae and erosion formation. Multiple subtypes of pemphigus disease have been identified based on their distinct clinical features and pathophysiology, including pemphigus vulgaris (PV), pemphigus foliaceus (PF), IgA pemphigus, and paraneoplastic pemphigus (PNP). The significant morbidity and mortality associated with pemphigus disorders warrants a review of their pathogenesis, clinical presentation, and diagnostic work-up. Assessment of standard and new therapies builds further conviction in the evaluation and management of these rare bullous dermatoses.

Pemphigus occurs worldwide but has a disproportionate geographic and ethnic distribution, with a significantly higher prevalence in patients of Ashkenazi Jewish or Mediterranean descent [1,2,3,4]. Despite its increased prevalence in these populations, pemphigus universally affects all racial and ethnic groups [3,4,5]. The prevalence of pemphigus disease in Ashkenazi Jewish populations may be attributed to the increased presence of several distinct HLA class II genes, specifically HLA-DRB1*04 and HLA-A*10 [6,7,8]. Overall, the epidemiologic trends associated with pemphigus diseases largely vary based on region of the world and the ethno-demographic characteristics of the population being studied [1,2,3,6,7].

Pemphigus largely affects patients between the ages of 50 and 60, although the mean age at diagnosis can differ significantly based on the country of origin and ethnic background. In some Western Asian nations such as Kuwait, the mean age at diagnosis is 36.5 years, whereas in European nations such as Bulgaria, the mean age at diagnosis is 72.4 years [9].

Importantly, disease onset in the pediatric population has also been described, including in patients as young as 6 years old [10]. Although the diagnosis of pemphigus in younger patient populations has increasingly been identified in recent studies, pemphigus disease is very rare in children overall. The global male-to-female ratio of pemphigus patients is approximately equal. Nonetheless, adolescent girls are more likely to be affected than adolescent boys [11,12].

## 2. Pathophysiology

Central to the pathogenesis of pemphigus is the presence of immunoglobulin (Ig) antibodies against proteins on the cell surface of keratinocytes. Previous studies incorporating immunochemical and molecular cloning laboratory techniques have demonstrated that the antigenic targets in PV and PF are desmogleins, transmembrane glycoproteins associated with desmosomes that confer cell-cell adhesion within the epidermis [4,13,14]. Notably, desmosomes are the most prevalent cell-cell adhesion junction proteins in the stratified squamous epithelium. There are additional adhesive protein complexes that play a similar role as intercellular support connections, such as adherens junctions, but desmosomes are unique in their ability to mediate strong, longitudinal adhesion that supersedes the weaker ability of adherens junctions [13].

Desmogleins are members of the cadherin supergene family, which categorizes PV and PF as cadherin autoimmune diseases. Antigens involved in PV have been identified as desmogleins 1 and 3, whereas PF solely involves desmoglein 1 [14,15]. The amino-terminal portion of desmoglein antigens has been identified as the most pathogenic epitope, given that IgG antibodies, directed against the amino-terminal portion of desmoglein 3, but not the carboxy-terminal portion, induce the formation of epithelial blisters [13]. IgG antibodies, directed against desmogleins, disrupt the adhesive function of desmosomes and impede their ability to function in standard cell-cell adhesion, resulting in epidermal acantholysis and flaccid blister formation, the characteristic clinical feature of pemphigus diseases.

The localization of blister formation and involvement of mucosal surfaces varies with the pemphigus disease subtype and can be explained by the desmoglein compensation theory. This theory states that, in cutaneous surfaces, desmoglein 1 is primarily expressed throughout all layers of the epidermis, while desmoglein 3 is primarily expressed in the deeper layers. In mucous membranes, the expression of desmoglein 1 is minimal while desmoglein 3 predominates [15]. The interpretation of the desmoglein compensation theory, as it relates to the clinical findings in pemphigus, can be summarized as follows: patients with antibodies against only desmoglein 3 should have mucosal-dominant PV because desmoglein 1 compensates for the loss of desmoglein 3 in skin. In the mucous membranes, desmoglein 3 is predominantly expressed; the low levels of desmoglein 1 cannot offset the inhibition of desmoglein 3, leading to epithelial acantholysis and mucosal erosions. When antibodies develop against both desmogleins 1 and 3, epidermal acantholysis occurs in both the skin and mucous membranes. Although the desmoglein compensation theory is still widely discussed in literature, subsequent data has raised questions about whether this concept sufficiently explains the complex pathogenic mechanism of pemphigus [14,15].

Patients with PF develop antibodies directed exclusively against desmoglein 1 and present with superficial blistering and cutaneous erosions without mucosal involvement [15]. In contrast, patients with PV develop antibodies against desmoglein 3 with or without targeting desmoglein 1. Positivity for desmoglein 3 antibodies results in mucosal-only PV, while positivity for desmoglein 1 and 3 antibodies results in mucocutaneous PV. Compared to PF, the acantholysis of PV affects the deeper parts of the epidermis, resulting in clinically deeper erosions.

IgA pemphigus is categorized into the subcorneal pustular dermatosis (SPD) type and the intraepidermal neutrophilic dermatosis (IEN) type. The target antigen in the SPD type of IgA pemphigus is desmocollin 1, another desmosomal cadherin protein [16]. The IEN type involves IgA antibodies directed against desmogleins 1 and 3. A common underlying pathophysiologic origin, linking all forms of IgA pemphigus, has not yet been elucidated [16,17].

Several distinct antigenic targets have been identified in the pathogenesis underlying PNP. These antigens are cytoskeletal attachment proteins, known as plakins, and include desmoplakin I, bullous pemphigoid antigen I, envoplakin, desmoplakin II, and periplakin [18]. Although not a plakin, alpha-2-macroglobulin-like-1 has also been identified as a characteristic antigen involved in PNP. Epitope spreading can also lead to the development of antibodies against desmocollins and desmogleins 1 and 3. These antibodies develop as a result of a paraneoplastic process, most commonly due to non-Hodgkin lymphoma, chronic lymphocytic leukemia, and Castleman disease [19].

## 3. Clinical Features

### 3.1. Pemphigus Vulgaris

A universal hallmark of the PV subtype of pemphigus disease is mucosal involvement in the form of painful blisters and erosions that predominate in the oropharyngeal mucous membranes. More than 90 percent of patients with PV present with mucous membrane involvement, and the oral cavity represents the most common site of mucosal lesions in PV patients, as well as the site in which initial disease manifestations are most likely to appear [20]. Although PV largely presents within the oral cavity, mucous membrane involvement can extend to include other mucosal sites, such as the eyes, nose, esophagus, vulva, vagina, cervix, and anus. Thus, the clinical presentation of PV can include ocular irritation, dysphagia, vocal hoarseness, vaginal irritation, and dyspareunia; in cervical disease, the histopathology of PV can be mistaken for dysplasia in Papanicolaou smears [4,20]. Mucosal erosions can be overwhelmingly painful for some patients and can cause daily tasks, such as chewing and eating, to become increasingly difficult as the disease progresses. This can subsequently result in poor nutrition, weight loss, and fatigue.

**Figure 1:**
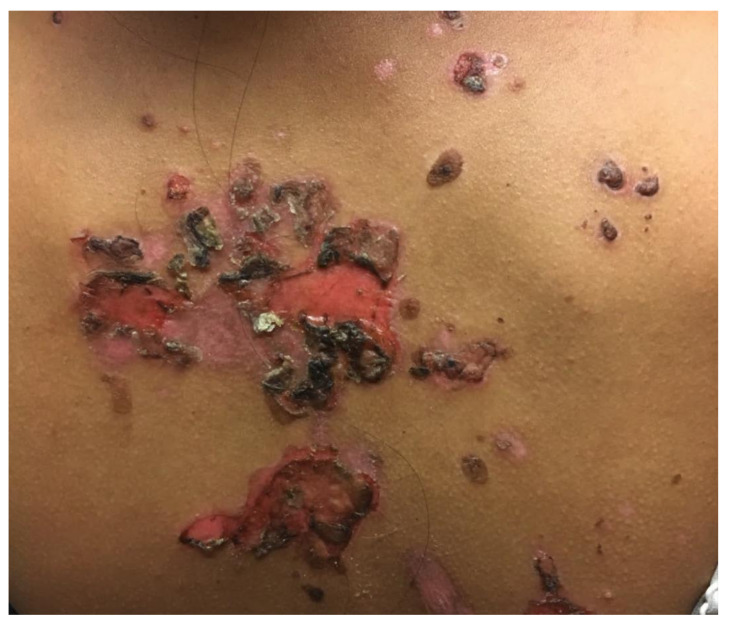
Pemphigus vulgaris. Flaccid bullae and crusted erosions on the back.

**Figure 2:**
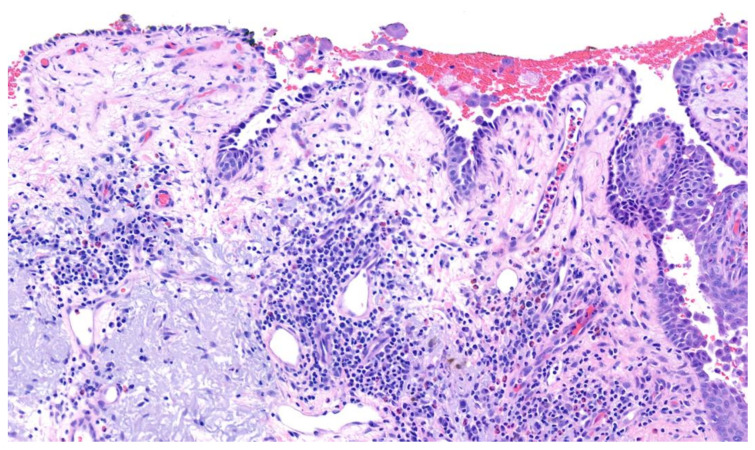
Pemphigus vulgaris. Suprabasilar acantholysis with follicular involvement and ‘tombstoning’ of basilar keratinocytes.

PV involves two main subgroups: the mucosal-dominant type, which produces mucosal erosions, but has minimal skin involvement, and the mucocutaneous type, which produces diffuse mucosal involvement in addition to cutaneous blisters and erosions. Cutaneous lesions of PV typically reflect flaccid blisters and crusted erosions (Figure 1) on an erythematous base [20,21]. The flaccid nature of the blisters seen in PV are secondary to the intraepidermal acantholysis (Figure 2) caused by anti-desmoglein antibodies. PV lesions are often Nikolsky sign-positive, signifying that mechanical pressure applied to a blister with little force results in shearing of adjacent skin. Cutaneous lesions can either be localized or diffuse and can affect any surface. Commonly, the lesions are excruciatingly painful. Importantly, the palms and soles are typically spared in patients with PV. The paucity of palmoplantar involvement can be helpful in distinguishing PV from other vesiculobullous dermatoses, such as PNP or erythema multiforme.

PV represents a chronic, long-lasting condition that can be adequately controlled with medical therapy. The prognosis of PV has improved drastically over the decades as a result of recent advancements in corticosteroid and steroid-sparing treatments [21,22,23]. Despite these advances in treatment, death still occurs in select patients with pemphigus, largely due to secondary infection. If left untreated, PV is typically fatal as a result of bacterial and viral infections and fluid and electrolyte imbalances [23]. Most patients who are left untreated ultimately die within the first few years following diagnosis. Without adequate treatment, the reported mortality of PV is greater than 75% [22].

Morbidity and mortality of PV is related to several factors, including the extent of mucocutaneous involvement, the dose of corticosteroid treatment required for treatment, and high-risk comorbidities. The prognosis of patients with PV is generally worse in elderly or immunocompromised patients and in those with extensive or severe disease [23]. Patients who are diagnosed at <65 years of age are likely to fare better than those who are diagnosed at >65 years of age [23].

Pemphigus vegetans is a rare, localized form of PV and represents one of the rarest subtypes of pemphigus disease overall [24]. Pemphigus vegetans is characterized by vegetating plaques that resemble cauliflower and are typically identified in flexural and intertriginous areas. This subtype of PV is mainly seen in middle-aged adults with mean age at diagnosis of 40–60 years. Although lesions are primarily evidenced in flexural areas, vegetations may manifest anywhere. As with its parent disorder, PV, pemphigus vegetans typically involves the mucous membranes [21,24].

### 3.2. Pemphigus Foliaceus

**Figure 3:**
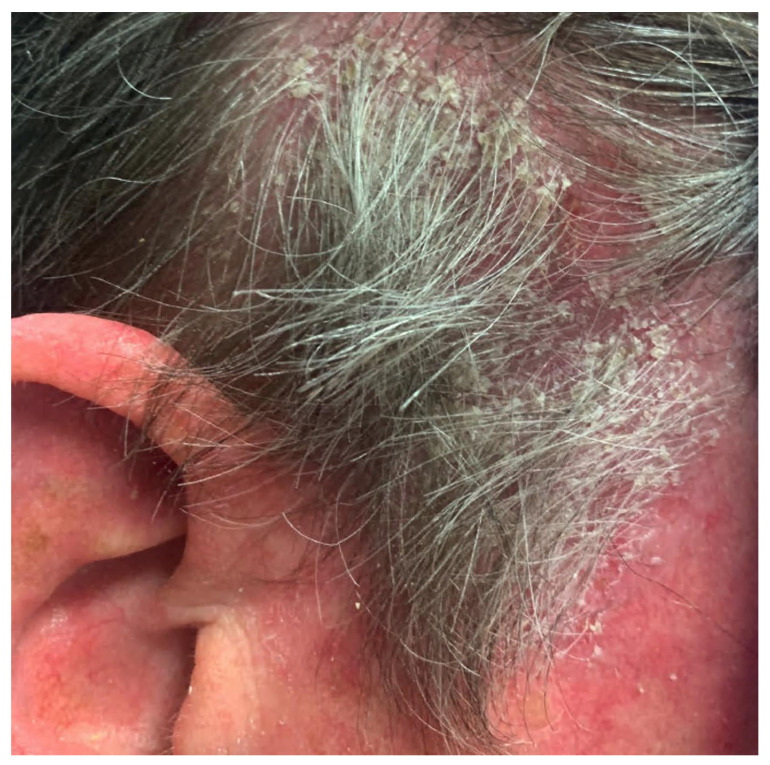
Pemphigus foliaceus. Thin erosions with bran-like scale on the scalp.

**Figure 4:**
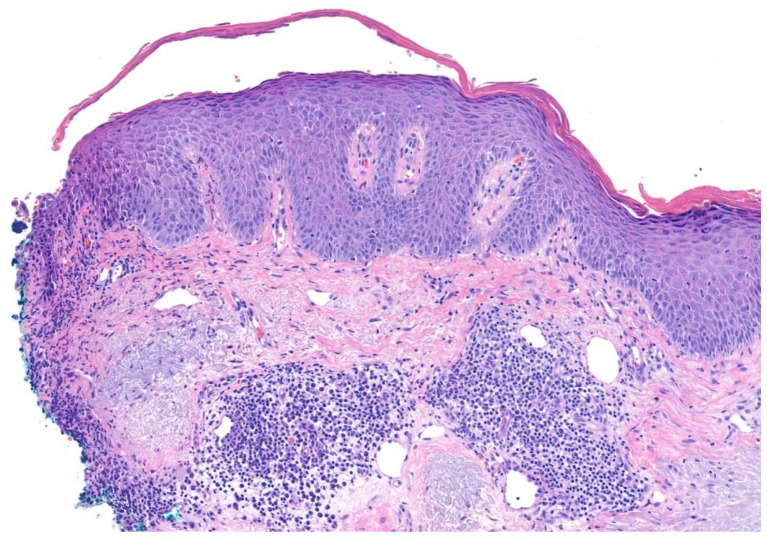
Pemphigus foliaceus. Superficial acantholysis within the granular layer.

PF represents a superficial variant of pemphigus that is caused by antibodies against desmoglein 1. Patients with PF typically present with cutaneous lesions without mucosal involvement [23]. This results in the lack of extracutaneous symptoms seen in PV, such as ocular pain, dysphagia, vocal hoarseness, or dyspareunia. Seborrheic areas of involvement predominate in PF, including the scalp, face, and upper trunk. Cutaneous lesions in PF characteristically involve scattered superficial blisters that devolve into crusted erosions on an erythematous base. These thin, delicate crusted erosions have been described as “bran-like” or resembling “cornflakes” (Figure 3). Similar to patients with PV, patients with PF typically present with skin fragility and are Nikolsky sign-positive [25]. Classically, erosions in PF are more superficial than that in PV, due to subcorneal acantholysis (Figure 4) compared to intraepidermal acantholysis, but this is not always apparent clinically. Cutaneous lesions in PF can progress beyond a standard seborrheic distribution to diffusely involve larger areas of skin in severe cases. Patients with PF often complain of pain or a burning sensation in areas where skin lesions are present, although symptoms are typically milder than those seen in PV. Systemic symptoms, such as fever, nausea, or vomiting, are usually absent [25].

Subtypes of PF include endemic PF (fogo selvagem), which presents with clinical symptoms similar to the idiopathic form of the disease, but it is linked to an environmental or endemic source (i.e., black flies (Simulium species)), and pemphigus erythematosus (Senear-Usher syndrome), which describes a localized variant of PF with a malar distribution reminiscent of the “butterfly” rash of systemic lupus erythematosus [26,27].

Drug-induced pemphigus can occur as PF or PV secondary to medication use. Drug-induced pemphigus can arise days to months following initiation of medication. One of the most common causes of drug-induced pemphigus is exposure to thiol drugs, such as penicillamine or captopril [28]. Thiol-induced pemphigus commonly manifests as drug-induced PF, whereas non-thiol drugs are more likely to trigger drug-induced PV. Overall, drug-induced pemphigus is more likely to present as PF rather than PV in a 4:1 ratio [29].

### 3.3. Paraneoplastic Pemphigus

Paraneoplastic pemphigus (PNP), also known as paraneoplastic autoimmune multiorgan syndrome (PAMS), represents a clinically heterogeneous autoimmune bullous dermatosis that occurs secondary to an underlying neoplasm. The most commonly associated neoplastic diseases associated with PNP are, in decreasing order of frequency, non-Hodgkin lymphoma, chronic lymphocytic leukemia, Castleman disease, thymoma, retroperitoneal sarcomas, and Waldenström macroglobulinemia [28,30]. Patients with PNP typically have severe mucosal involvement, polymorphous skin lesions, an associated neoplasm, and pulmonary involvement with features of bronchiolitis obliterans. Chronic erosive, and painful, mucositis serves as the pillar for PNP diagnosis, and diagnosis should not be made without this key clinical feature [28].

Patients with PNP typically suffer from cutaneous lesions after the onset of mucosal lesions. The morphology of these lesions has variable presentations, including bullous pemphigoid-like, pemphigus-like, erythema multiforme-like, lichen planus-like, and graft-versus-host disease-like. Large areas of desquamation may mimic toxic epidermal necrolysis [30,31,32]. Because the cutaneous presentation of PNP can be variable, clinical suspicion must be high to coordinate the appropriate diagnostic work-up.

Progressive respiratory failure, with clinical features of bronchiolitis obliterans, is often cited as the most common cause of mortality in patients with PNP [28,30,33,34]. Possible causes of PNP-associated respiratory failure include infection, toxic effects induced by chemotherapy, neoplasia, and autoantibody-mediated pulmonary injury. Deposits of IgG in the bronchial epithelium, which have been observed in patients with PNP, suggest that autoantibody-mediated injury may play a distinct role in this process [34,35]. The frequency of bronchiolitis obliterans respiratory disease, in patients with PNP, is largely undetermined, but one study detected pulmonary involvement in 26 of 28 patients [33,36].

Recently updated diagnostic criteria, based on literature analysis, identified three major and two minor criteria for diagnosis of PNP. Meeting all three major criteria or two major and both minor criteria fulfill a diagnosis of PNP. Major criteria include (a) mucositis with or without cutaneous involvement, (b) concomitant internal neoplasm, and (c) serologic evidence of anti-plakin antibodies; minor criteria include (a) acantholysis and/or lichenoid interface dermatitis on histopathology and (b) direct immunofluorescence staining, showing intercellular and/or basement membrane staining [37].

The prognosis associated with PNP is largely unfavorable with a 75% to 90% mortality rate [38,39,40,41]. Mortality associated with PNP can be attributed in part to the underlying malignancy that plays a central role in its pathogenesis.

### 3.4. IgA Pemphigus

IgA pemphigus is a rare subtype of pemphigus disease defined by IgA antibodies that target transmembrane adhesion proteins in the epidermis. The underlying pathophysiology has been linked to various autoimmune and inflammatory malignancies and disease states, including HIV infection, Sjogren syndrome, rheumatoid arthritis, and inflammatory bowel disease. However, key aspects of its etiology remain undefined [17,42]. IgA pemphigus is further subdivided into two types of pemphigus disease: (1) SPD type, which primarily manifests with antibodies concentrated in the upper epidermis (targeting desmocollin 1), and (2) IEN type, which manifests with antibodies concentrated throughout the epidermis (targeting desmogleins 1 and 3). Despite their distinct antigenic targets, both types of IgA pemphigus are clinically characterized by vesiculopustular lesions.

When compared to PV, patients with IgA pemphigus present with a milder clinical course. Skin lesions in patients with IgA pemphigus initially appear as tense vesicles on erythematous bases that later transform into pustules [17,42]. A recent study found that the most common manifestations of IgA pemphigus were vesicles (81%), pustules (75%), and erythematous annular plaques (64%) [17]. Patients with IgA pemphigus often develop circinate plaques with herpetiform vesicles. About half of patients suffer from pruritus, and many complain of pain associated with blisters [17,42].

The most frequently affected cutaneous sites, in patients with IgA pemphigus, include the flexural areas of the proximal extremities and the trunk; however, the scalp, postauricular areas, and intertriginous areas may also be affected. Importantly, mucous membranes are typically spared in patients with IgA pemphigus, although oral mucosal and perianal involvement has been reported in a small number of patients [43]. Most often, patients diagnosed with IgA pemphigus are not affected by systemic symptoms such as fever or weight loss.

## 4. Diagnosis

A summary of the clinical, histopathological, and serological findings of the various pemphigus disorders is outlined in Table 1.

Diagnosis of pemphigus begins with a thorough history and physical exam. During the history, clinicians should ascertain the presence of mucosal involvement, as the presence of mucosal lesions can differentiate subtypes of pemphigus disease. PV and PNP always involve the mucosa, while PF and IgA pemphigus typically do not. Importantly, mucosal involvement in pemphigus disease can be inconspicuous, and mucosal surfaces routinely visible during standard physical exams, such as the eyes and lips, may not be involved. For example, a patient with PV may present with hoarseness and dysphagia secondary to occult mucosal involvement of the posterior oropharynx. Therefore, clinicians should be sure to evaluate for ocular symptoms, hoarseness of voice, dysphagia, and dyspareunia to assess for involvement of all mucosal surfaces.

Medications should be reviewed in detail. Clinical presentation and laboratory studies cannot reliably distinguish between idiopathic pemphigus and drug-induced pemphigus. Recent studies have demonstrated that thiol and phenol-based medications are most closely linked to drug-induced pemphigus. Some of the most common triggering medications involved in drug-induced pemphigus include penicillamine, captopril, tiopronin, aspirin, heroine, rifampin, levodopa, non-steroid anti-inflammatory drugs, and calcium channel blockers [29].

Following a thorough history and physical exam, the laboratory work-up of pemphigus disease includes at least two biopsies with or without serum collection for indirect immunofluorescence (IIF), enzyme-linked immunosorbent assay (ELISA), or immunoblotting [21,26]. A 4 mm lesional biopsy should be taken from the edge of an early lesion or erosion for hematoxylin and eosin (H&E) staining and routine histopathologic examination. An additional perilesional skin biopsy should be taken from normal-appearing skin, 4 mm from a vesicle or erosion, for direct immunofluorescence (DIF) [21,44]. Biopsies of lesional skin for DIF are more likely to be linked to false negative results as a result of the destruction of immunoreactants involved in the inflammatory process of the underlying pemphigus disease. Clinicians should also be sure to avoid placing DIF biopsies in formalin and instead utilize Michel medium, or Zeus medium, for adequate preservation. Serum is collected for ELISA and/or IIF to identify serologic evidence of pathogenic antibodies. The distinctive findings on histopathology, IIF, and ELISA/immunoblotting for each pemphigus disorder are depicted in Table 1.

## 5. Management

The treatment approach for pemphigus mainly relies on immune system suppression to prevent new lesion formation and heal existing bullous skin and/or mucous lesions, while minimizing serious treatment side effects. For all forms of pemphigus, the literature supports corticosteroids as a first-line therapy, with or without adjuvant therapies. Generally, mild pemphigus treatment involves lower steroid doses compared to moderate/severe disease treatment, which involves higher steroid doses with the addition of steroid-sparing adjuvant agents [21,26,45]. Historically, azathioprine and mycophenolate mofetil were first-line corticosteroid-sparing adjuvant therapies [45,46]. More recently, the chimeric anti-CD20 monoclonal antibody rituximab has become a treatment of choice, both at disease onset and especially for refractory disease [47,48,49]. Ultimately, for all forms, the most important factor is treating quickly and effectively and to taper corticosteroids to avoid recurrence [50]. Most treatments are considered successful if they improve disease significantly within the first two months, characterized by healed lesions without the appearance of new lesions. If successful, treatment should be tapered gradually, starting with the corticosteroids, followed by the adjuvant nonsteroidal agent [21,48]. On average, clinically significant improvement is expected on the timeline of weeks for PF and months for PV [21,48,50].

### 5.1. Pemphigus Vulgaris and Foliaceus

**Figure 5:**
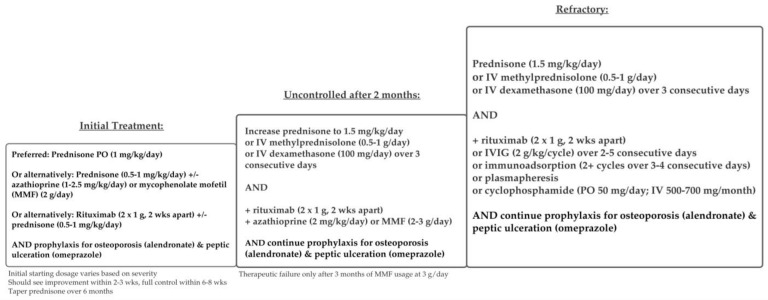
Treatment schema for pemphigus vulgaris.

**Figure 6:**
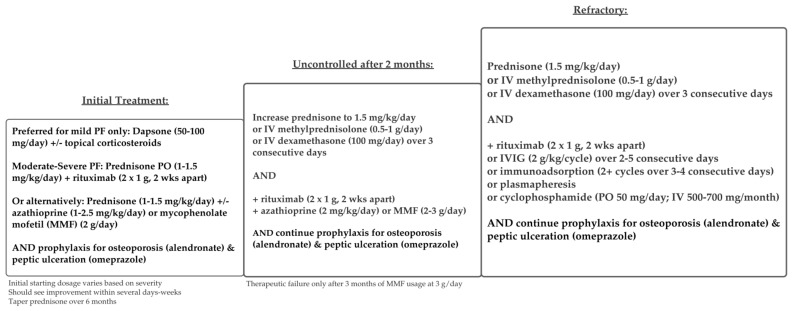
Treatment schema for pemphigus foliaceus.

Treatment regimens for PV and PF are similar and are depicted in Figure 5 and Figure 6. Although many studies combine both patient populations, some current guidelines recommend different treatments for mild PF versus mild PV [48].

Overall consensus from the literature supports systemic glucocorticoids as first line-treatment for all forms of PV, with or without adjuvant therapies. For mild PF, topical steroids, systemic steroids, dapsone, and rituximab are listed as first-line treatments, according to the updated 2020 guidelines from The European Academy of Dermatology and Venereology (EADV) [48]. For mild PV, systemic steroids and rituximab are first-line treatments. Treatment protocols for moderate-severe PV and PF are the same, according to these guidelines.

Guidelines differ in drug dosages and protocols according to disease severity. There are several disease severity scoring systems, but the most common are the Autoimmune Bullous Skin Disorder Intensity Score (ABSIS), which accounts for skin and oral involvement, the amount of body surface area (BSA) involved, and severity as defined by subjective discomfort while doing activities of daily living (ADL), and the Pemphigus Disease and Area Index (PDAI) score, which assesses severity by the blisters’ anatomical locations (cutaneous versus mucosal) and the amount of damage [48,49,51]. The EADV guidelines separate specific treatment dosages by disease severity, as measured by the amount of body surface area (BSA) affected and/or by the PDAI score [48,49]. According to EADV guidelines, mild PV is defined as BSA < 5% and/or PDAI score < 15. Moderate PV is defined as BSA > 5% and/or PDAI score > 15 but <45. Severe PV is defined as PDAI score > 45 [48]. Protocols differ in starting dosages for rituximab and prednisone [48]. British guidelines differ slightly in that they recommend separating PV treatment into two stages: an initial remission induction and maintenance of remission; initial first-line therapy depends on corticosteroids alone, with the addition of various adjuvant therapies to maintain remission [21].

The addition of adjuvant agents allows for lower initial steroid dosages, leading to fewer adverse effects and better overall outcomes [21,48]. The most effective one is rituximab, a monoclonal anti-CD20 antibody [46,51,52]. Although typically used as an adjuvant agent, some studies have used rituximab to replace systemic corticosteroids as first-line therapy [53,54]. Rituximab has also been indicated (alone or with corticosteroids) as first-line for mild PV, according to EADV guidelines [21], and is also an effective maintenance therapy [55]. Rituximab was approved for PV in 2018 by the United States Food and Drug Administration [56]. However, rituximab can be cost prohibitive ($990 for every 10 mg/mL) [57], and thus, it is not an accessible initial treatment for all patients.

Alternative adjuvant agents commonly used include mycophenolate mofetil [58,59], cyclophosphamide [60,61,62], and azathioprine [60,61]. Azathioprine and mycophenolate mofetil are commonly used in lieu of rituximab, due to cost [57,61], or when there are contraindications to rituximab [22,53]. Refractory pemphigus is difficult to treat, and there are many case studies supporting the use of various adjunct agents such as intravenous immune globulin (IVIg), immunoadsorption, or plasmapheresis, as well as other supportive treatments, including steroid mouthwashes and analgesic sprays to treat oral erosions [62,63].

A multidisciplinary approach is necessary for pemphigus patients due to its chronic relapsing course. Thus, it is important to consider prophylactic medications to prevent further complications [48]. Osteoporosis protocol is important to initiate at the start of treatment due to the use of long-term corticosteroids. Histamine blockers and/or proton pump inhibitors should be considered to prevent peptic ulcers. Prophylaxis for pneumocystis pneumonia (PCP), caused by *Pneumocystis jiroveci*, is not routinely indicated for pemphigus patients, despite prolonged use of immunosuppressive therapies and overall increased risk of opportunistic infections [64,65]. A final consideration is that patients cannot receive live vaccines if treated with rituximab or other adjuvant immunosuppressants [48]. Further research is needed to delineate which agents are most effective with minimal side effects.

### 5.2. IgA Pemphigus

**Figure 7:**
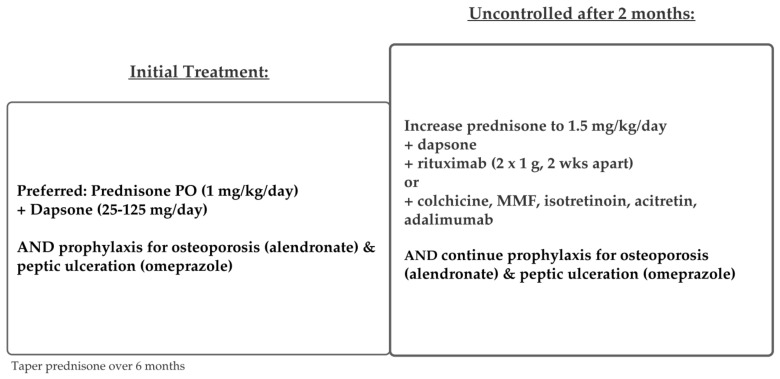
Treatment schema for IgA Pemphigus.

There are currently no consensus treatment guidelines for IgA pemphigus [16]. IgA pemphigus is not well controlled by corticosteroids alone, but it typically follows a milder and more limited course than IgG-mediated pemphigus. Current literature supports adding dapsone to systemic corticosteroids as first-line therapy [66]. Dapsone is thought to help suppress the neutrophilic infiltration central to the pathogenesis of this disease. Other potentially effective treatments include colchicine, mycophenolate mofetil, isotretinoin, acitretin, and adalimumab, as illustrated in Figure 7 [67].

### 5.3. Paraneoplastic Pemphigus

**Figure 8:**
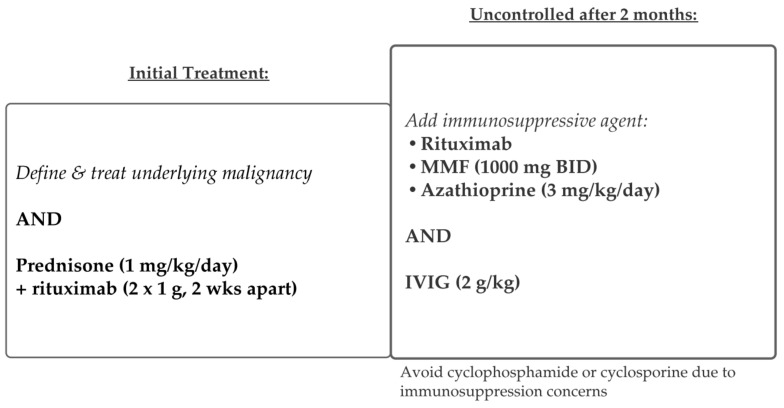
Treatment schema for paraneoplastic pemphigus.

PNP occurs in association with a variety of malignancies, most commonly lymphoproliferative diseases [30,34,35]. In most patients (2/3), PNP is diagnosed after the underlying neoplastic disorder [38,63]. Because PNP is rare, most treatment data is limited to case reports. Thus, there are no definitive guidelines for management. The treatment success for PNP depends, mainly, on prompt diagnosis and early treatment of the patient’s underlying malignancy. Thus, the first step in therapy is identifying, staging, and treating the associated neoplasm in PNP. Concurrent treatment with standard corticosteroid therapy, with or without other immunosuppressive agents, is necessary in most cases [30,35]. Rituximab is often used in PNP, although the response to therapy is variable (Figure 8) [37]. Refractory PNP is particularly difficult to treat; various monoclonal antibodies have been used successfully in case reports, including alemtuzumab (anti-CD52 monoclonal antibody) [63]. Daclizumab (anti-CD25 monoclonal antibody) was another potentially promising therapy for PNP before being recalled in 2018 [41,42]. Symptom management is also critical: antiseptic mouthwashes and narcotic pain medications are sometimes necessary [40]. Despite various forms of treatment, PNP remains more resistant to treatment than all other forms of pemphigus and has the highest mortality rate (75–90%), with most patients dying from sepsis, malignancy, or respiratory failure [18,41].

## 6. Conclusions

Pemphigus is a family of rare autoimmune bullous dermatoses that affects the skin and mucous membranes. PV and PF are classically characterized by flaccid bullae that correlate with the histopathologic finding of acantholysis. IgA pemphigus displays pustular lesions, clinically, that parallel similar findings under light microscopy. PNP is a paraneoplastic process that has highly variable clinical and histopathologic findings, although the uniting factor is the evidence of anti-plakin antibodies. This family of diseases is characterized by a profound morbidity for patients, and before the advent of corticosteroid therapy, overwhelming mortality. Therapeutic options, such as anti-CD20 therapy, have improved the prognosis of patients with pemphigus and decreased the morbidity associated with conventional immunosuppression.

## Figures and Tables

**Table 1:** Clinical diagnosis and laboratory work-up of pemphigus diseases; hematoxylin and eosin (H&E), direct immunofluorescence (DIF), indirect immunofluorescence (IIF).

	Clinical Presentation	H&E	DIF	IIF	Serology
**Pemphigus vulgaris**	Painful blisters and erosions predominating in oropharyngeal mucous membranes; symptoms can include dysphagia, vocal hoarseness, vaginal irritation, painful sexual intercourse; palms and soles are spared	Suprabasilar acantholysis with retention of basal keratinocytes along the basement membrane (“tombstoning”)	Intercellular deposition of immunoglobulin G (IgG)	Intercellular deposition of immunoglobulin G (IgG) circulating antibodies *Utilize monkey esophagus substrate*	Presence of antibodies against both desmoglein 1 and desmoglein 3 *or* antibodies against desmoglein 3 only
**Pemphigus foliaceus**	Painful blisters and erosions without mucosal membrane involvement; cutaneous involvement primarily concentrated in seborrheic areas (scalp, face, upper trunk)	Acantholysis within upper epidermis, adjacent or within the granular layer, leading to a subcorneal cleft *If significant eosinophils are present, consider drug-induced pemphigus*	Intercellular deposition of immunoglobulin G (IgG) *Negative DIF studies are not uncommon in drug-induced pemphigus*	Intercellular deposition of immunoglobulin G (IgG) circulating antibodies *Utilize guinea pig esophagus substrate*	Presence of antibodies against desmoglein 1 only
**Paraneoplastic pemphigus**	Severe mucosal involvement and polymorphous skin lesions with associated underlying malignancy or neoplasm (e.g. Non-Hodgkin’s lymphoma, chronic lymphocytic leukemia, Castleman’s disease)	Suprabasilar acantholysis, keratinocyte necrosis, and interface change	Intercellular deposition of immunoglobulin G (IgG) *Negative DIF studies are not uncommon in paraneoplastic pemphigus*	Intercellular deposition of immunoglobulin G (IgG) circulating antibodies *Utilize rat bladder substrate*	Presence of antibodies against plakin proteins
**IgA pemphigus**	Tense bullae that transition into clear fluid-filled blisters; cutaneous involvement of vesicles (81%), pustules (75%), and erythematous annular plaques (64%) primarily seen in flexural areas of proximal extremities and trunk; mucous membranes usually spared	Subcorneal pustular dermatosis type: subcorneal vesiculopustules with minimal acantholysis Intraepidermal neutrophilic dermatosis type: intraepidermal vesiculopustules with variable acantholysis	Intercellular deposition of immunoglobulin A (IgA)	Intercellular deposition of immunoglobulin A (IgA) circulating antibodies	Subcorneal pustular dermatosis type: presence of antibodies against desmocollin 1 Intraepidermal neutrophilic dermatosis type: presence of IgA antibodies against desmoglein 1 and 3
